# Alteration of PD-L1 expression and its prognostic impact after concurrent chemoradiation therapy in non-small cell lung cancer patients

**DOI:** 10.1038/s41598-017-11949-9

**Published:** 2017-09-12

**Authors:** Daichi Fujimoto, Keiichiro Uehara, Yuki Sato, Ichiro Sakanoue, Munehiro Ito, Shunsuke Teraoka, Kazuma Nagata, Atsushi Nakagawa, Yasuhiro Kosaka, Kojiro Otsuka, Yukihiro Imai, Hiroshi Hamakawa, Yutaka Takahashi, Masaki Kokubo, Keisuke Tomii

**Affiliations:** 1 Department of Respiratory Medicine, Kobe City Medical Center General Hospital, Kobe, Japan; 2 Department of Clinical Pathology, Kobe City Medical Center General Hospital, Kobe, Japan; 3 Department of Thoracic Surgery, Kobe City Medical Center General Hospital, Kobe, Japan; 4 Department of Radiation Oncology, Kobe City Medical Center General Hospital, Kobe, Japan

## Abstract

Concurrent chemoradiation therapy (CCRT) is the treatment of choice for locally advanced non-small cell lung cancer (LA-NSCLC). Several clinical trials that combine programmed cell death 1 (PD-1) axis inhibitors with radiotherapy are in development for patients with LA-NSCLC. However, the effect of CCRT on programmed cell death ligand-1 (PD-L1) expression on tumor cells is unknown. In this study, we analysed paired NSCLC specimens that had been obtained pre- and post-CCRT. PD-L1 expression on tumor cells was studied by immunohistochemistry. A total of 45 patients with LA-NSCLC were included, among which there were sufficient pre- and post-CCRT specimens in 35 patients. Overall, the percentage of tumor cells with PD-L1 expression significantly decreased between pre- and post-CCRT specimens (P = 0.024). Sixteen, 15, and 4 patients had decreased, unchanged, or increased PD-L1 expression after CCRT, respectively. Median OS of patients with decreased, unchanged, or increased PD-L1 expression was 85.1, 92.8, and 14.6 months, respectively (P < 0.001). In conclusion, the percentage of PD-L1-positive tumor cells significantly decreased after CCRT. Alteration of PD-L1 expression after neoadjuvant CCRT was associated with prognosis in patients with LA-NSCLC. These data should be considered when developing the optimal approach of integrating PD-1 axis inhibitors with CCRT.

## Introduction

Lung cancer is the leading cause of cancer-related deaths worldwide^1^. Non-small-cell lung cancer (NSCLC) accounts for approximately 80% of lung cancers, and concurrent chemoradiation therapy (CCRT) is the treatment of choice for locally advanced NSCLC (LA-NSCLC)^2^. However, the prognosis of LA-NSCLC remains poor, despite recent efforts to improve outcomes via the use of new cytotoxic drugs or high-dose radiotherapy^2–4^.

Recently, programmed cell death 1 (PD-1)/programmed cell death ligand-1 (PD-L1) checkpoint inhibitors demonstrated impressive anti-tumor activity for the treatment of metastatic NSCLC^5–9^. Thus, there is substantial interest in extending the benefit of these inhibitors to LA-NSCLC patients. Although there are limited data on the efficacy of combining radiation therapy and immunotherapy, this combination has the ability to achieve a synergistic therapeutic effect^10–12^. Several clinical trials that combine these agents with radiotherapy in patients with LA-NSCLC are in the planning stage^13, 14^. Therefore, we need the optimal approach to integrate PD-1 axis inhibitors with CCRT.

In most trials of PD-1 axis inhibitors for metastatic NSCLC, immunohistochemical (IHC) analysis of PD-L1 expression has been used as a predictive diagnostic test to identify responders and to guide treatment in trials of PD-1 axis inhibitors in NSCLC patients^5–9^. Particularly, in a recent first-line trial, pembrolizumab was associated with significantly longer progression-free and overall survival than platinum-based chemotherapy in patients with advanced NSCLC and PD-L1 expression on at least 50% of tumor cells. Thus, high PD-L1 expression is a potential good predictive biomarker for the efficacy of PD-1 axis inhibitors. Despite the successful use of PD-L1 expression in the trial, there were several problems in assessing PD-L1 expression. One problem was that the expression of PD-L1 on tumor cells was not consistent. Anti-cancer systemic therapy influenced PD-L1 expression on tumor cells in previous reports^15, 16^. However, the effect of CCRT on PD-L1 expression on tumor cells is not known.

The aims of this study were to analyse paired NSCLC specimens that had been obtained pre- and post-CCRT to explore the impact of CCRT on PD-L1 expression and to suggest possible optimal approaches of integrating PD-1 axis inhibitors with CCRT.

## Results

### Patient characteristics

**Figure 1:**
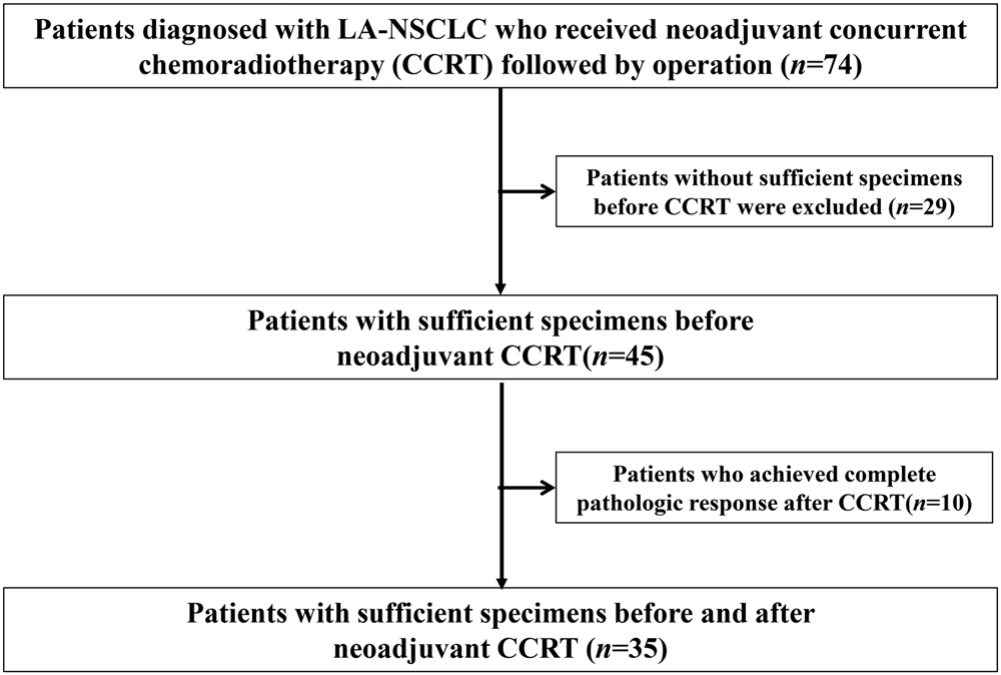
Patient selection and exclusion criteria.

A total of 45 LA-NSCLC patients with sufficient specimens before CCRT were included in this study (Fig. 1). All patients were treated with CCRT followed by surgery. Patient characteristics are summarized in Table 1. The median time between the last induction day of CCRT and the surgery was 32 days (interquartile range, 29–36 days). Fourteen patients had stage II disease, while 31 patients had stage III disease. The majority of patients (67%) received vinorelbine plus platinum as the CCRT regimen. Only 4 patients received adjuvant chemotherapy after the surgery. Twenty-eight (62%) had positive PD-L1 expression on tumor cells in the pre-CCRT specimens.

**Table 1:** Characteristics of patients with pre-CCRT specimens.

Patient characteristic	Total (%) (N = 45)
Age (years)	
Mean (SD)	63.9 (9.8)
Sex	
male	36 (80)
Smoking status	
never-smoker	6 (13)
current or former smoker	39 (87)
Histology	
adenocarcinoma	19 (42)
squamous	24 (53)
others	2 (5)
Stage	
II A	6 (13)
II B	8 (18)
III A	30 (67)
III B	1 (2)
ECOG-PS	
0	26 (58)
1	19 (42)
Chemo-radiotherapy regimen	
Vinorelbine plus platinum	30 (67)
Docetaxel plus platinum	10 (23)
Paclitaxel plus platinum	3 (6)
S1 plus platinum	1 (2)
Pemetrexed plus platinum	1 (2)
Radiotherapy dose	
40 Gy	38 (84)
50 Gy	7 (16)
Down staging *	
Yes	32 (71)
No	13 (29)
PD-L1 status on tumor cells before CCRT	
positive	28 (62)
negative	17 (38)
CD8+ lymphocytes density before CCRT**	
high	0 (0)
intermediate	14 (33)
low	29 (67)

SD, standard deviation; ECOG PS, Eastern Cooperative Oncology Group Performance Status; PD-L1, programmed cell death ligand-1; CCRT, concurrent chemo-radiation therapy.

*Pathological stage compared with clinical stage.

**We had sufficient material with stroma and tumor cells before CCRT in 43 patients.

### PD-L1 status and CD 8+ lymphocytes density between matched pre- and post-CCRT specimens

**Figure 2:**
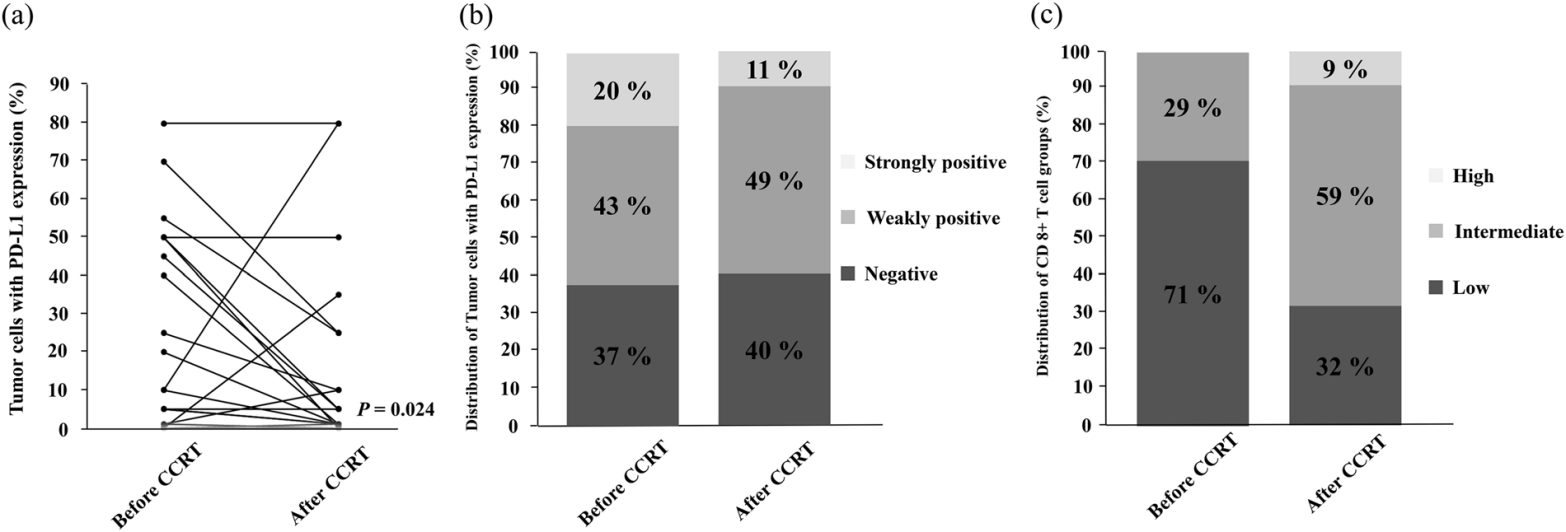
Comparison of the level of PD-L1 expression on tumor cells in the pre- and post-CCRT specimens (**a** and **b**), and comparison of the number of stromal CD8+ lymphocytes (low, intermediate, high) in the pre- and post-CCRT specimens (**c**).

Among the 45 patients with sufficient material before CCRT, there were 35 patients in whom there was sufficient material with tumor cells both before and after CCRT (Table 2). Among the 35 patients, 22 patients (63%, 22/35) had PD-L1 expression on tumor cells in the pre-CCRT specimens, and 21 patients (60%, 21/35) had PD-L1 expression on tumor cells in the post-CCRT specimens. Overall, the percentage of tumor cells with PD-L1 expression significantly decreased between the pre- and post-CCRT specimens (P = 0.024) (Fig. 2a and b). Sixteen, 15, and 4 patients had decreased, unchanged, or increased PD-L1 expression after CCRT compared with that before CCRT, respectively. Of the 15 patients with unchanged PD-L1 expression, PD-L1 expression was negative in both the pre-CCRT specimens and it remained negative in the post-CCRT specimens in 11 patients (73%, 11/15).

**Table 2:** Characteristics of the 35 patients with both pre- and post-CCRT specimens.

Patient characteristic	Total (%) (N = 35)
Age (years)	
Mean (SD)	62.6 (10.3)
Sex	
male	28 (80)
Smoking status	
never-smoker	5 (14)
current or former smoker	30 (86)
Histology	
adenocarcinoma	16 (46)
squamous	18 (51)
others	1 (3)
Stage	
II A	6 (17)
II B	8 (23)
III A	20 (57)
III B	1 (3)
ECOG-PS	
0	26 (74)
1	9 (26)
Chemo-radiotherapy regimen	
Vinorelbine plus platinum	25 (71)
Docetaxel plus platinum	5 (14)
Paclitaxel plus platinum	3 (9)
S1 plus platinum	1 (3)
Pemetrexed plus platinum	1 (3)
Radiotherapy dose	
40Gy	31 (89)
50Gy	4 (11)
Down staging *	
Yes	22 (63)
No	13 (37)
PD-L1 status on tumor cells before CCRT	
positive	22 (63)
negative	13 (37)
PD-L1 status on tumor cells after CCRT**	
positive	21 (60)
negative	14 (40)
Change in PD-L1 expression level on tumor cells after CCRT **	
increased	4 (11)
unchanged	15 (43)
decreased	16 (46)
CD8+ lymphocytes density before CCRT***	
high	0 (0)
intermediate	10 (29)
low	24 (71)
CD8+ lymphocytes density after CCRT **	
high	3 (9)
intermediate	20 (57)
low	12 (34)
Change in CD8+ lymphocytes density after CCRT ****	
increased	17 (50)
unchanged	16 (47)
decreased	1 (3)

SD, standard deviation; ECOG PS, Eastern Cooperative Oncology Group Performance Status; PD-L1, programmed cell death ligand-1; CCRT, concurrent chemo-radiation therapy.

*Pathological stage compared with clinical stage.

**We had sufficient material with tumor cells before and after CCRT in 35 patients.

***We had sufficient material with stroma and tumor cells before CCRT in 34 patients.

****We had sufficient material with stroma and tumor cells before and after CCRT in 34 patients.

We compared the clinical profiles of the patients with decreased (n = 16) or non-decreased PD-L1 expression (n = 19) after CCRT. The patients with decreased PD-L1 expression included a significantly lower proportion of patients with increased CD8+ lymphocytes density (3/16 vs. 14/18, respectively, P < 0.001; one patient with non-decreased PD-L1 expression did not have sufficient material with stroma) (Table 3).

**Table 3:** Characteristics of the patients with decreased or non-decreased PD-L1 expression on tumor cells between pre- and post-CCRT specimens.

Patient characteristics	Decreased (%) (N = 16)	Not decreased (%) (N = 19)	P-value
Age (years)			
Mean (SD)	61.1 (10.4)	63.9 (10.4)	0.428
Sex			
male	13 (81)	15 (79)	0.865
female	3 (19)	4 (21)	
Smoking status			
never-smoker	1 (6)	4 (21)	0.349
current or former smoker	15 (94)	15 (79)	
Histology			
squamous	7 (44)	11 (58)	0.406
Non-squamous	9 (56)	8 (42)	
Stage			
II	6 (38)	8 (42)	0.782
III	10 (62)	11 (58)	
Chemo-radiation therapy regimen			
Vinorelbine based	11 (69)	14 (74)	0.748
Taxan based *	3 (19)	5 (26)	0.594
Radiotherapy dose			
40Gy	13 (81)	18 (95)	0.206
50Gy	3 (19)	1 (5)	
Down staging			
Yes	11 (69)	11 (58)	0.507
No	5 (31)	8 (42)	
Change in stromal CD8+ lymphocyte status after CCRT **			
Increased	3 (19)	14 (78)	<0.001
Unchanged or decreased	13 (81)	4 (22)	

CCRT, concurrent chemo-radiation therapy; SD, standard deviation.

*Including docetaxel and paclitaxel.

**One patient was excluded because this case did not have enough material with stroma.

We also investigated the changes in stromal CD8+ lymphocytes density after CCRT. Sufficient material with stroma and tumor cells before CCRT was available in 43 of the 45 patients. Further, we had sufficient material with stroma and tumor cells both before and after CCRT in 34 of the 35 patients; there was not enough material with stroma in the pre-CCRT specimens of one patient. Among the 34 patients with sufficient material with tumor cells and stroma before and after CCRT, one, 16, or 17 patients had decreased, unchanged, or increased stromal CD8+ lymphocytes after CCRT, respectively (Fig. 2c).

### Association of PD-L1 expression with survival time

**Figure 3:**
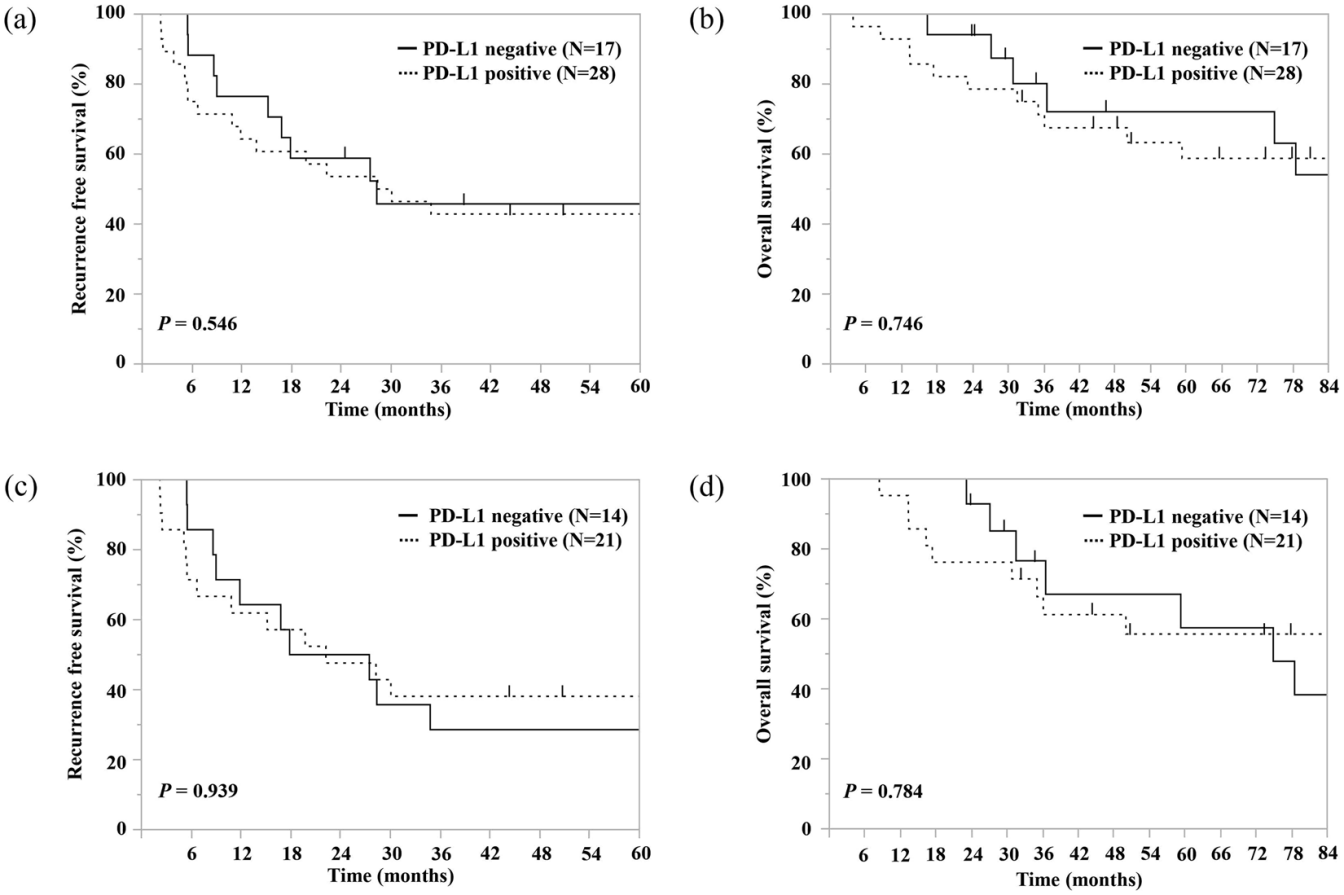
Kaplan–Meier survival curves. Kaplan–Meier survival curves of overall survival in patients with or without PD-L1 expression on tumor cells in the pre-CCRT specimens (**a**), or post-CCRT specimens (**b**). Kaplan–Meier survival curves of recurrence-free survival in patients with or without PD-L1 expression on tumor cells in the pre-CCRT specimens (**c**), or post-CCRT specimens (**d**).

Among the 45 patients with sufficient pre-CCRT material with tumor cells, PD-L1 expression in pre-CCRT material was not significantly associated with RFS (PD-L1-positive group versus PD-L1-negative group, median 28.8 versus 27.9 months, p = 0.546, respectively) and OS (PD-L1-positive group versus PD-L1-negative group, median 94.1 versus 92.8 months, p = 0.746, respectively). Among the 35 patients with sufficient post-CCRT tumor material, PD-L1 expression in post-CCRT material was not significantly associated with RFS (PD-L1-positive group versus PD-L1-negative group, median 21.9 versus 22.4 months, p = 0.939, respectively) and OS (PD-L1-positive group versus PD-L1-negative group, median 85.1 versus 73.9 months, p = 0.784, respectively). These survival curves are shown in Fig. 3.

**Figure 4:**
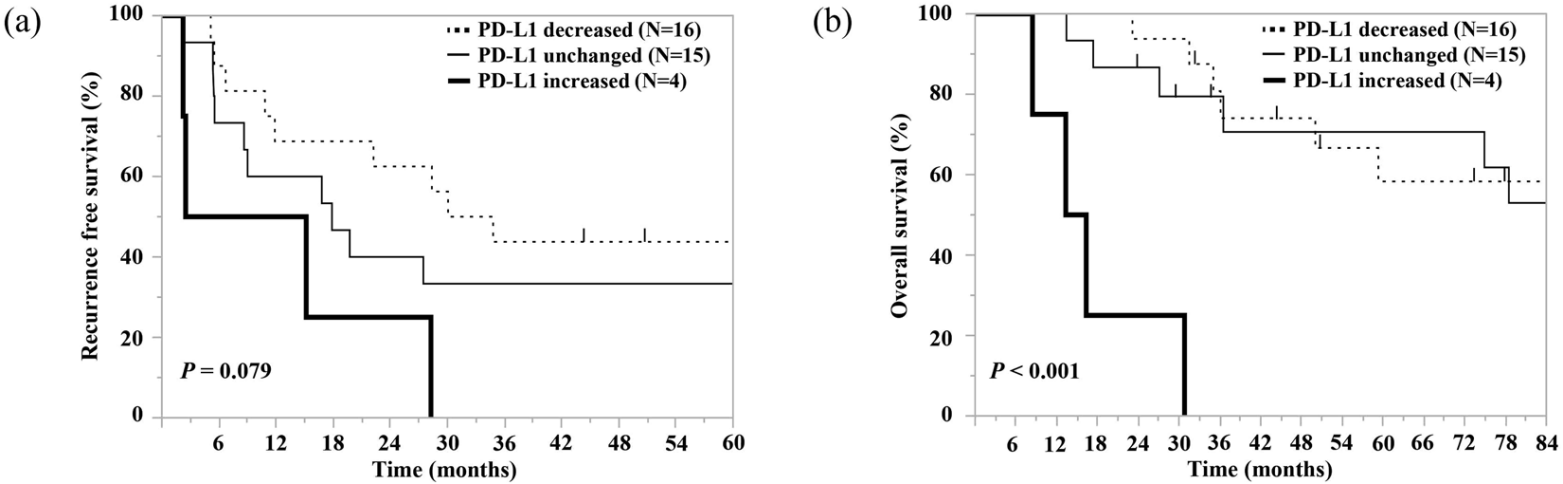
Kaplan–Meier survival curves of recurrence-free survival (**a**) and overall survival (**b**) in patients with decreased, unchanged, or increased PD-L1 expression between the pre- and post-CCRT specimens.

We also investigated the association between the change in PD-L1 expression and survival time. The median RFS of patients with decreased, unchanged, or increased PD-L1expression was 32.0, 17.6, and 8.7 months, respectively (P = 0.079). In addition, the median OS of patients with decreased, unchanged, or increased PD-L1expression was 85.1, 92.8, and 14.6 months, respectively (P < 0.001). These survival curves are shown in Fig. 4. No patient received PD-1 axis inhibitors during the observation period.

### Association of CD8+ lymphocytes density with survival time

The patients with intermediate-high CD8+ lymphocytes density in the pre-CCRT material tended to have longer RFS than those with low CD8+ lymphocytes density in the pre-CCRT material (intermediate-high group versus low group of pre-CCRT material, median 94.0 versus 19.5 months, p = 0.127). The patients with intermediate-high CD8+ lymphocytes density in the post-CCRT material also tended to have longer RFS (intermediate-high group versus low group of post-CCRT material, median 27.1 versus 11.2 months, p = 0.093). In addition, there was a similar trend between CD8+ lymphocytes density and OS in patients with pre-CCRT material (intermediate-high group versus low group of pre-CCRT material, median not reached versus 77.4 months, p = 0.232) and patients with post-CCRT material (intermediate-high group versus low group of post-CCRT material, median 92.8 versus 35.6 months, p = 0.101). The change in CD8+ lymphocytes density was not significantly associated with RFS (increased group versus unchanged or decreased group, median 19.5 versus 21.9 months, p = 0.435, respectively) and OS (increased group versus unchanged or decreased group, median 92.8 versus 58.5 months, p = 0.786, respectively). These survival curves are shown in Supplementary Figure 2 and Supplementary Figure 3.

## Discussion

To the best of our knowledge, this study provides the first report of alteration of PD-L1 expression after neoadjuvant CCRT and its prognostic impact in patients with LA-NSCLC.

Our results indicated that the percentage of PD-L1-positive tumor cells significantly decreased after CCRT. Patients with increased PD-L1 expression after CCRT were relatively rare (4/35) in our study. A recent study also demonstrated that chemotherapy significantly reduced PD-L1 expression on tumor cells in NSCLC patients^15^. When we considered these results, CCRT can reduce PD-L1 expression on tumor cells in many patients.

In our study, we could not demonstrate the prognostic value of the baseline PD-L1 expression status, which still remains controversial in lung cancer^17^. Although we additionally analysed this prognostic association under the positive cut-off value of 5% of tumor cells, we could not demonstrate the prognostic value of the baseline PD-L1 expression status (data not shown). However, the change in PD-L1 expression after CCRT was associated with survival. Especially, increased PD-L1 expression was related to poor survival, although the proportion of patients with increased PD-L1 expression was small. A similar trend was reported in the neoadjuvant chemotherapy setting^15, 18^. The precise reason for this association was unclear. One possible explanation is the malignant feature of tumor cells with increased PD-L1 expression after anti-cancer treatment. One study reported that increased PD-L1 expression promoted the resistant response in lung cancer cells^18^. Another study indicated that high PD-L1 expression was related to high proliferative activity and the epithelial-mesenchymal transition phenotype^19^. The present study was too small to be able to draw firm conclusions. However, when we considered these previous results with ours, there seems to be an association between the change in PD-L1 expression and prognosis.

We could not find predictive factors associated with the change in PD-L1 expression in our study. Especially, the expression profile was not correlated with the chemotherapy regimen, although previous reports indicated the association *in vitro* in lung cancer tumor cells^20, 21^. There was a discrepancy in the effect of chemotherapy on PD-L1 regulation among different malignancies and agents^22–25^. In addition, a recent study demonstrated that chemotherapy decreased PD-L1 expression in clinical specimens of gastrointestinal cancers, although the same chemotherapy upregulated PD-L1 expression *in vitro* on tumor cells^26^. Therefore, future studies on this association using clinical lung cancer specimens are necessary.

We also found that the stromal CD8+ lymphocytes density increased after CCRT. We observed that patients with intermediate-high stromal CD8+ lymphocytes density in the pre- or post-CCRT material tended to have longer RFS and OS, similar to previous studies^27, 28^. However, the change in CD8+ lymphocytes density after CCRT was not associated with survival time. Interestingly, the patients with decreased PD-L1 expression included a significantly lower proportion of patients with increased CD8+ lymphocytes density. Increased PD-L1 expression and increased number of tumor-infiltrating lymphocytes were associated with better response to PD-1 axis inhibitors^9, 29, 30^. Therefore, these patients might have a good response to PD-1 axis inhibitors after CCRT.

Our study demonstrated the alteration of PD-L1 expression and its prognostic impact after CCRT in NSCLC patients. However, we did not investigate other immune checkpoint molecules such as PD-1 and cytotoxic T lymphocyte antigen-4 (CTLA-4) in our study. The up- and down-modulation of these molecules after radiation have also been reported in some studies^31, 32^. In a previous study in cervical cancer patients, elevated PD-1 expression on CD4+ T cells was demonstrated after CCRT^33^. Another study on rectal cancer indicated that CTLA-4 expression was relatively stable after chemoradiation therapy^34^. Actually, several small studies suggested the clinical efficacy of CTLA-4 inhibitor with radiation in several malignancies^35–37^. Although the prognostic importance of these molecules was not confirmed in clinical lung cancer studies, further research is warranted to study the association between chemoradiation and the change in the expression of these molecules in clinical specimens.

Several clinical trials that combine immune check point inhibitors with radiotherapy are in development for patients with LA-NSCLC^13, 14^. Therefore, we need the optimal approach to integrate PD-1 axis inhibitors with CCRT. Unfortunately, none of the patients in our cohort received PD-1/PD-L1 inhibitors after CCRT or combination therapy of PD-1/PD-L1 inhibitors and radiation. Therefore, these clinical studies are warranted in the future. We speculate two approaches in the treatment of LA-NSCLC, when we considered the results of our study. One approach is to administer PD-1 axis inhibitors before CCRT, because CCRT generally reduced PD-L1 expression. Another approach is to administer these agents after CCRT, because patients with increased PD-L1 expression had poor prognosis. In addition, patients with increased PD-L1 expression had increased CD8+ lymphocytes. Therefore, we might improve the prognosis of these poor prognostic patients by administering PD-1 axis inhibitors after CCRT.

This study had several limitations. First, there was the potential for selection bias, because this study was conducted at a single institution and we included patients who underwent surgery. Second, this study was limited by the relatively small sample size. However, even though the sample size was small, the main outcome was statistically significant. Therefore, we believe that this study provided evidence of decreased PD-L1 expression after CCRT and its associated increase in OS. Third, the chemotherapy regimen was determined by each doctor and therefore differed among the patients. Fourth, we did not investigate PD-L1 expression with another method such as fluorescent *in situ* hybridization (FISH).

In conclusion, CCRT significantly reduced the percentage of tumor cells with PD-L1 expression. Alteration of PD-L1 expression after neoadjuvant CCRT was associated with prognosis in patients with LA-NSCLC. We should consider these data to determine the optimal approach of integrating PD-1 axis inhibitors with CCRT in order to improve the prognosis of these patients.

## Methods

### Patients

We retrospectively analysed patients who were diagnosed with LA-NSCLC and who underwent CCRT followed by surgery (complete resection) at Kobe City Medical Center General Hospital between January 2004 and January 2013 (Fig. 1). We included patients in whom we had sufficient specimens with tumor cells before CCRT for analysis. This study included patients with stage II to III disease of LA-NSCLC who underwent surgery as part of a multimodality treatment approach^38^. Patients who reported never having smoked were defined as never smokers, those who had smoked within 1 year of the diagnosis were categorized as current smokers and the rest were considered to be former smokers. All patients were classified on the basis of clinical stage according to the 7th edition TNM classification^39^. The post-CCRT specimens were obtained at the time of surgical resection. Recurrence-free survival (RFS) was defined as the period from the day of surgery until any recurrence of lung cancer or death from any cause or the end of the follow-up. Overall survival (OS) was defined as the period from the day of surgery until death from any cause or the end of the follow-up. The observation period was until August 2016. This study was approved by the Kobe City Medical Center General Hospital Ethics Committee. All methods were performed in accordance with the 1975 Declaration of Helsinki. All tumor specimens in the pathological analysis were obtained with informed consent (or formal waiver of consent) with approval by the Ethics Committee of our hospital.

### Evaluation of PD-L1 expression and stromal CD8-positive T cells

Histological samples were defined as sufficient specimens if ≥100 tumor cells were present. The expression of PD-L1 in human NSCLC specimens was analysed by IHC staining using rabbit monoclonal anti-human antibody (clone 28-8, Abcam, 1:150). Four-μ m-thick sections were cut from the formalin-fixed, paraffin-embedded tumor block and then routinely deparaffinized and rehydrated. The antibody was applied according to the DAKO-recommended detection methods and previous reports^40–42^. A placenta specimen served as the positive control (Supplementary Fig. 1). The scoring of PD-L1 in tumor cells was expressed as a percentage of stained cells in the overall section of tumor and estimated in increments of 5% except for 1% positivity of tumor cells. Patients with 1% or greater PD-L1 staining of tumor cells were considered to be positive for PD-L1 expression, consistent with previous studies using this antibody^5–7^. Patients with positive PD-L1 expression were divided into two groups according to the main criteria commonly used in clinical NSCLC cases: weakly positive (percentage of stained cells of 1–49%), or strongly positive (percentage of stained cells of ≥50%)^43^. Unchanged PD-L1 expression was defined as the same percentage of stained cells in the specimens obtained before and after CCRT from the same patient.

The presence of stromal CD8-positive lymphocytes in NSCLC specimens was assessed with IHC staining using prediluted primary CD8 antibody from DAKO (C8/144B). The percentage of CD8-positive lymphocytes compared with the total amount of nucleated cells in the stromal compartments was assessed. The following cutoffs were used according to previous reports^44, 45^: low density ≤25%; intermediate density >25% to 50%; high density: >50%. An increase in CD8-positive lymphocytes was defined as a change from low density to intermediate or high density, or from intermediate to high density after CCRT.

Samples were anonymized and independently scored by two pathologists (K.U. and Y.I.). In cases of disagreement, the slides were re-examined and the two pathologists reached a consensus.

### Statistical analysis

Continuous variables were analysed using Student’s *t*-test. Dichotomous variables were analysed using the χ^2^ or Fisher’s exact test, as appropriate. The change in PD-L1 positivity status was analysed using Wilcoxon signed-rank test. The Kaplan–Meier method was used to estimate the survival outcomes and groups were compared using the log-rank test. The results are expressed as hazard ratios (HRs) with 95% confidence interval (CI). A P-value of < 0.05 was considered to indicate statistical significance. We conducted statistical analyses using JMP 9 software (SAS Institute, Cary, NC, USA).

## Electronic supplementary material

Supplementary Figure
