# Supplementary material for: Alteration of PD-L1 expression and its prognostic impact after concurrent chemoradiation therapy in non-small cell lung cancer patients
Source: Sci Rep. 2017 Sep 12;7:11373. doi: 10.1038/s41598-017-11949-9 (PMC5595796; doi:10.1038/s41598-017-11949-9)

Alteration of PD-L1 expression and its prognostic impact after concurrent chemoradiation therapy in non-small cell lung cancer patients

Daichi Fujimoto, Keiichiro Uehara, Yuki Sato, Ichiro Sakanoue, Munehiro Ito, Shunsuke Teraoka, Kazuma Nagata, Atsushi Nakagawa, Yasuhiro Kosaka, Kojiro Otsuka, Yukihiro Imai, Hiroshi Hamakawa, Yutaka Takahashi, Masaki Kokubo, Keisuke Tomii

Supplementary Figure 1. Immunohistochemical analysis of programmed cell death ligand-1 (PD-L1) expression in normal placenta (×40, positive control) (Figure 1A), and on tumor cells from patients with LA-NSCLC (×40; B: positive PD-L1 expression; C: negative PD-L1 expression). Representative specimens of a tumor invasive margin are shown (×40, D: hematoxylin eosin staining; E: IHC with CD8 antibody).

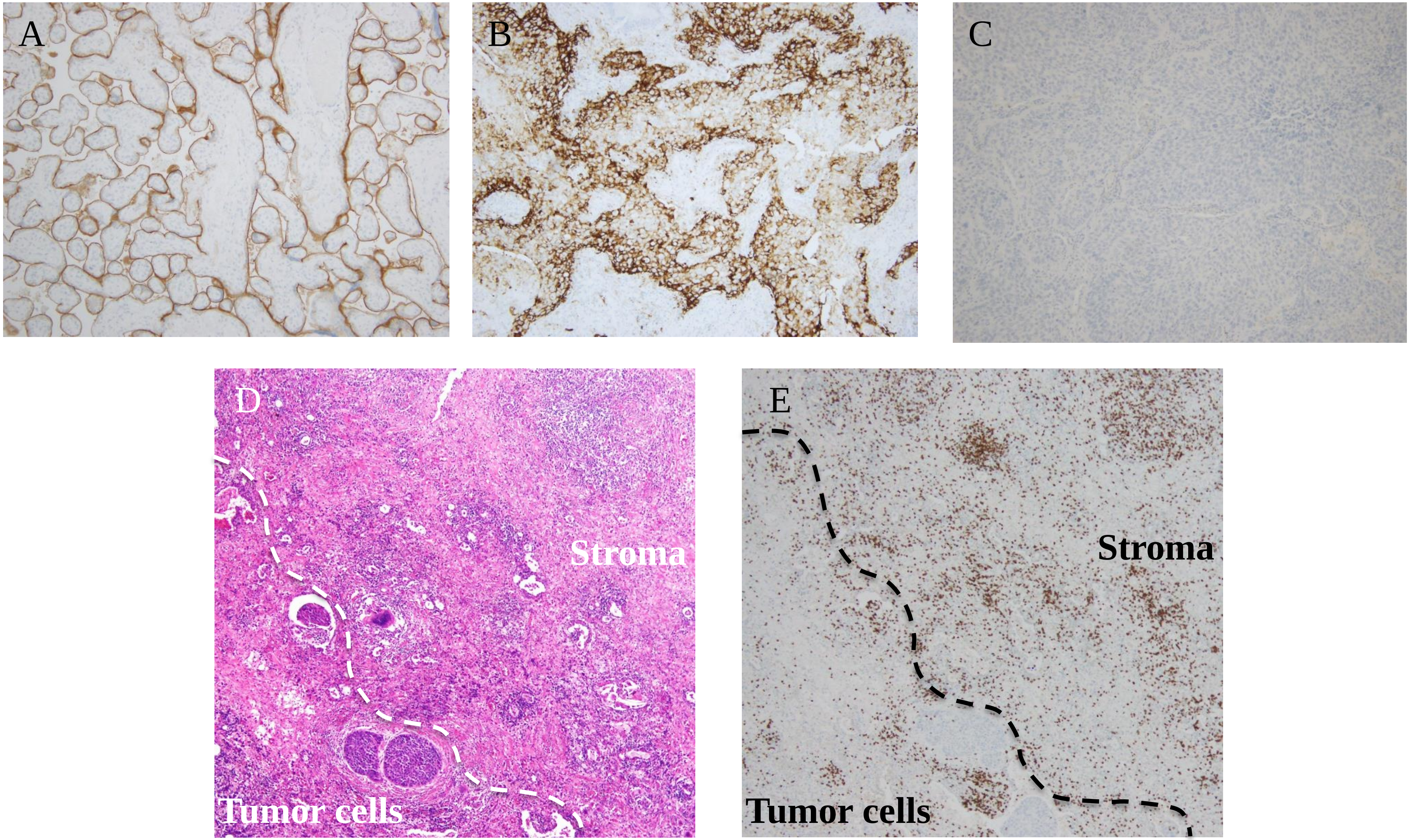

Supplementary Figure 2. Kaplan–Meier curves of recurrence-free survival (a) and overall survival (b) in patients with intermediate and high, or low number of CD8+ lymphocytes in pre-CCRT specimens, and in post-CCRT specimens (C and D, respectively).

Supplementary Figure 2a

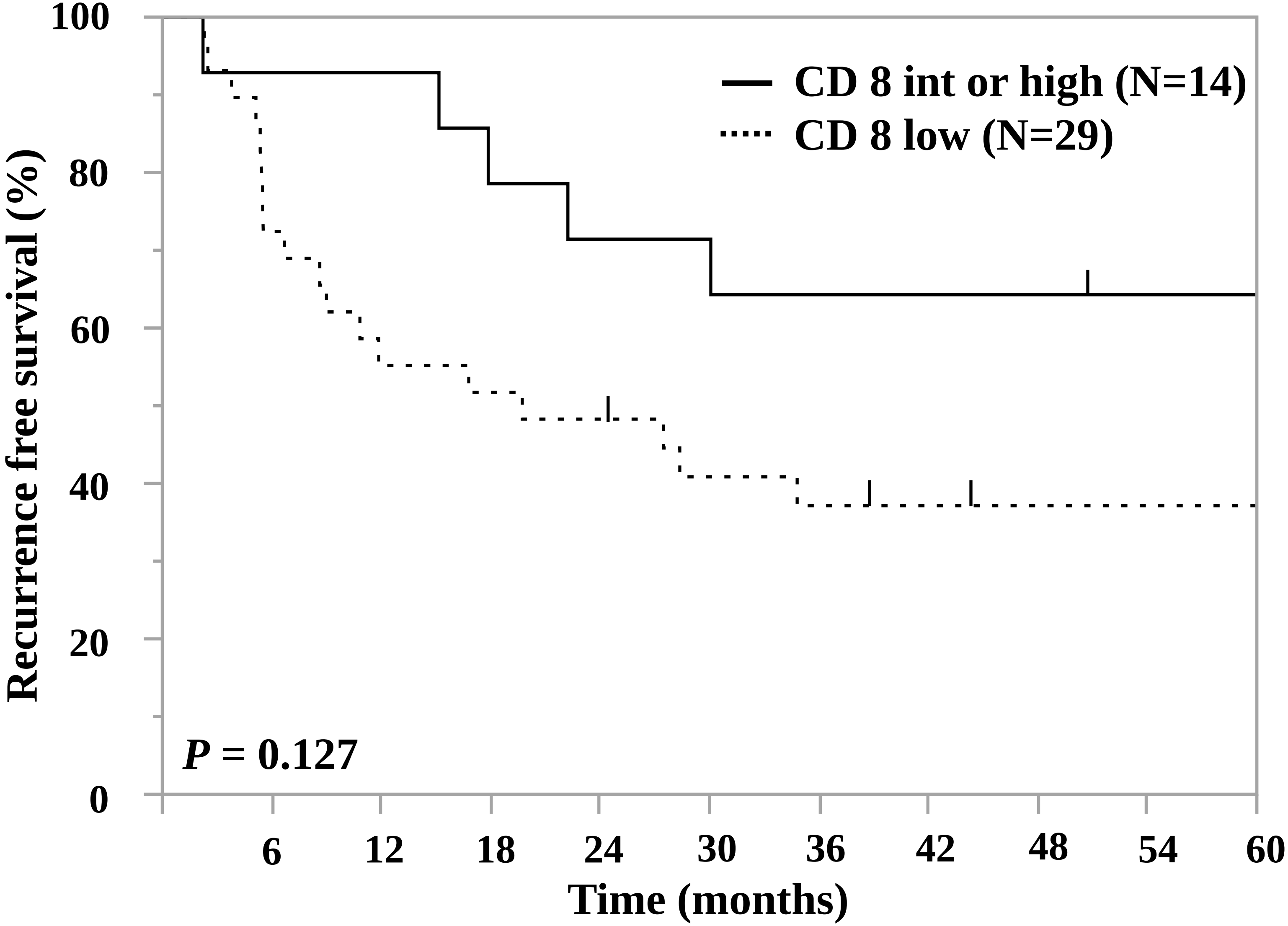

Supplementary Figure 2b

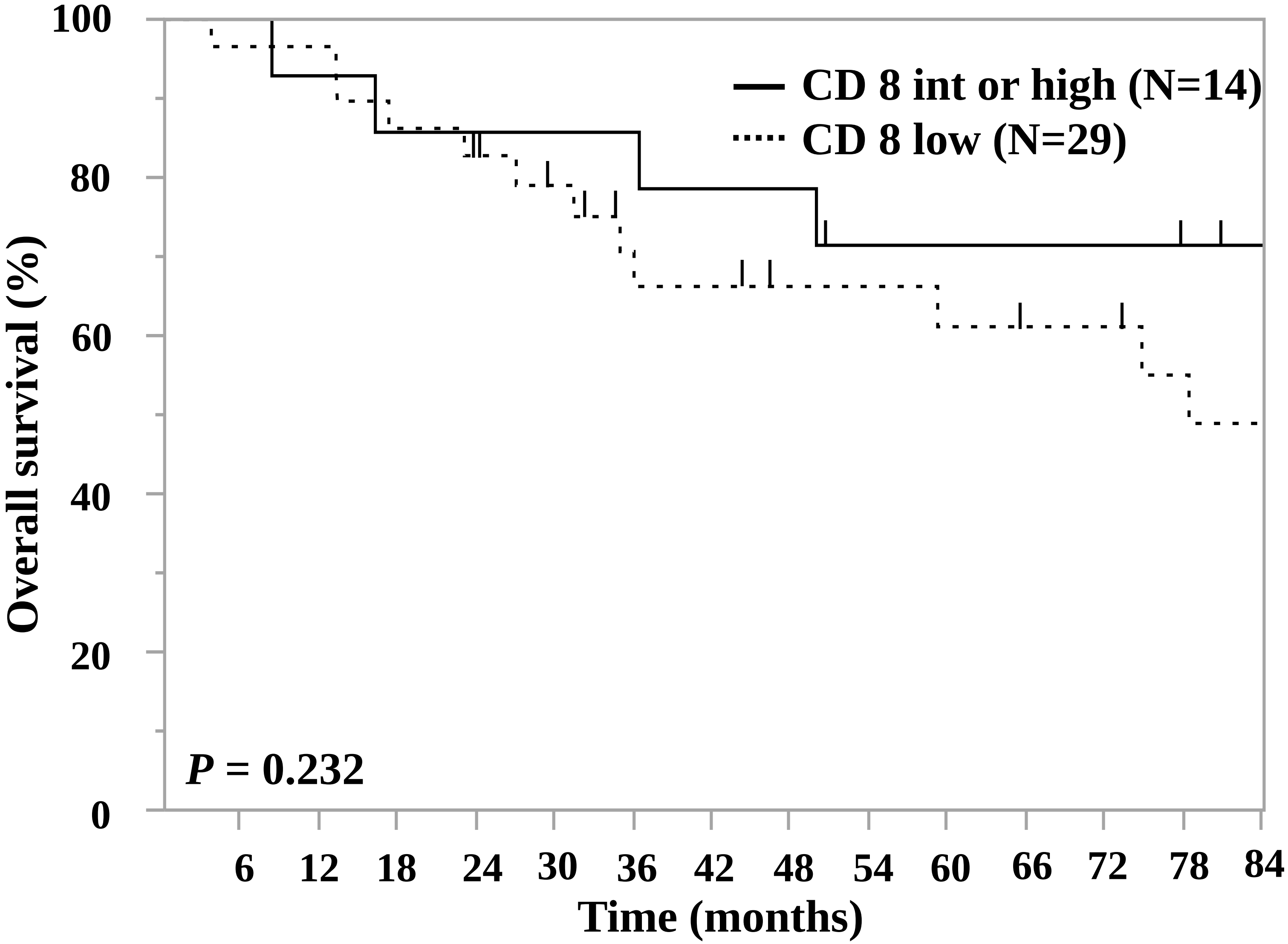

Supplementary Figure 2c

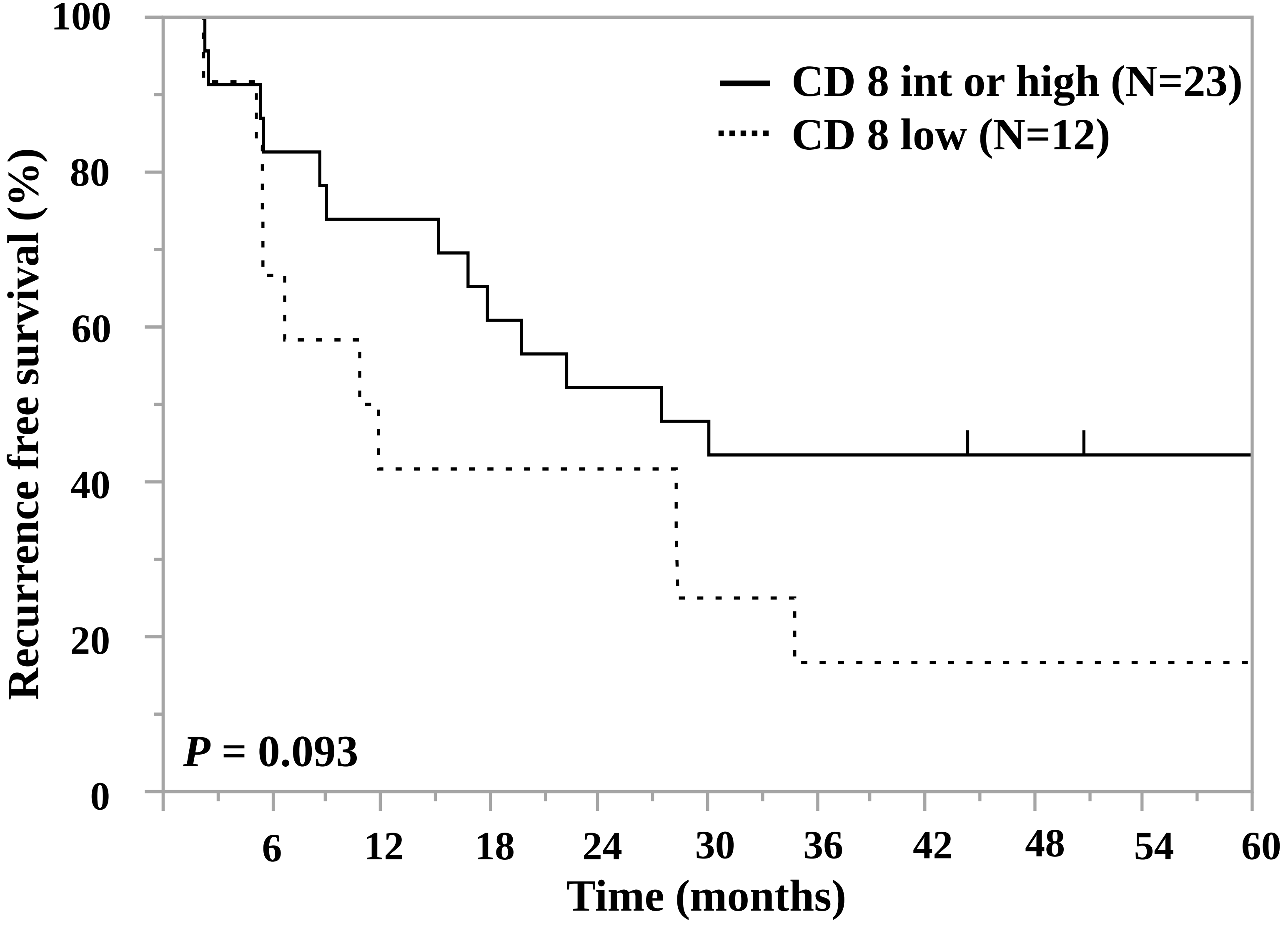

Supplementary Figure 2d

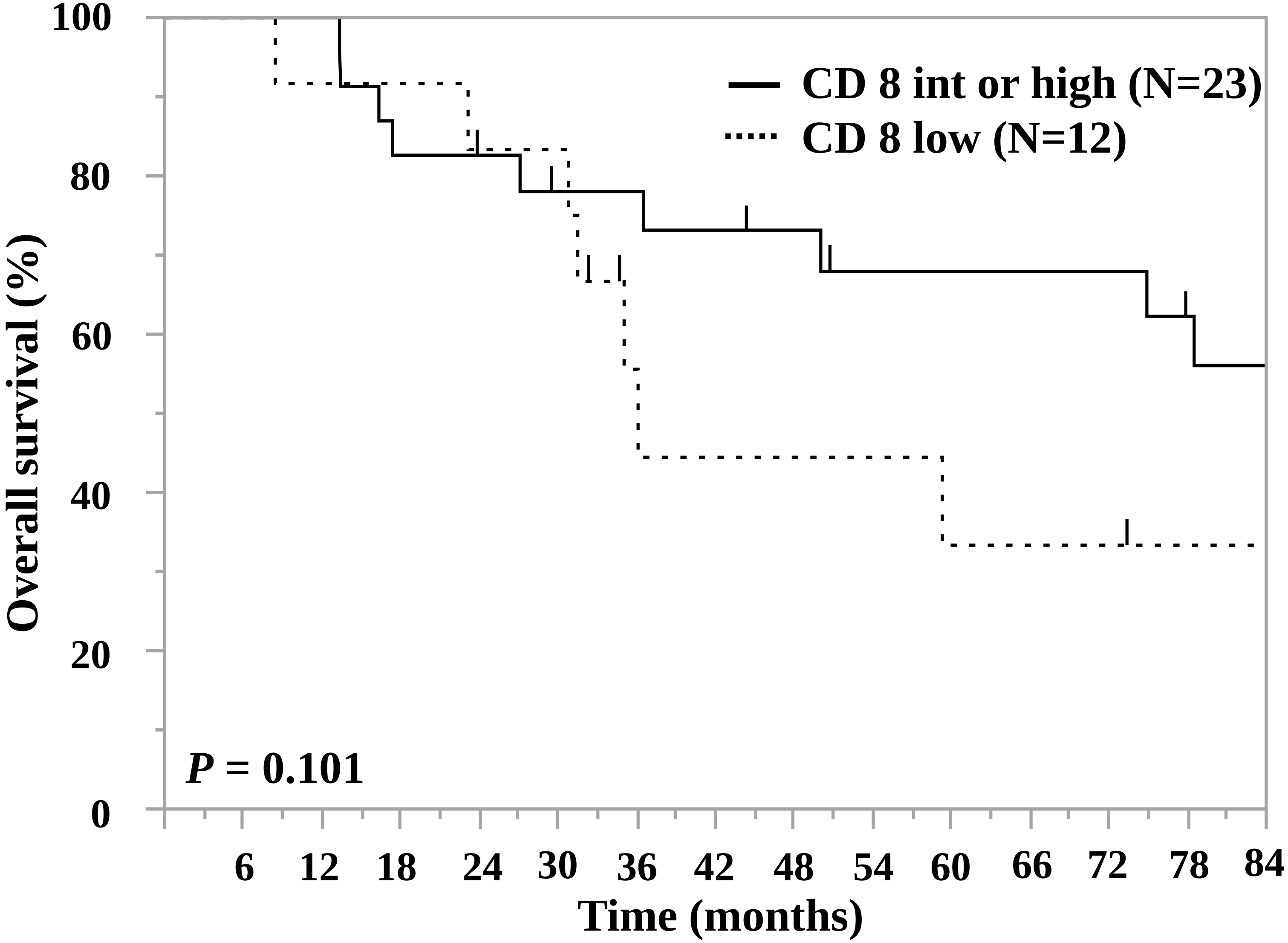

Supplementary Figure 3. Kaplan–Meier curves of recurrence-free survival (a) and overall survival (b) in patients with decreased and unchanged, or increased number of CD8-positive lymphocytes after CCRT.

Supplementary Figure 3a

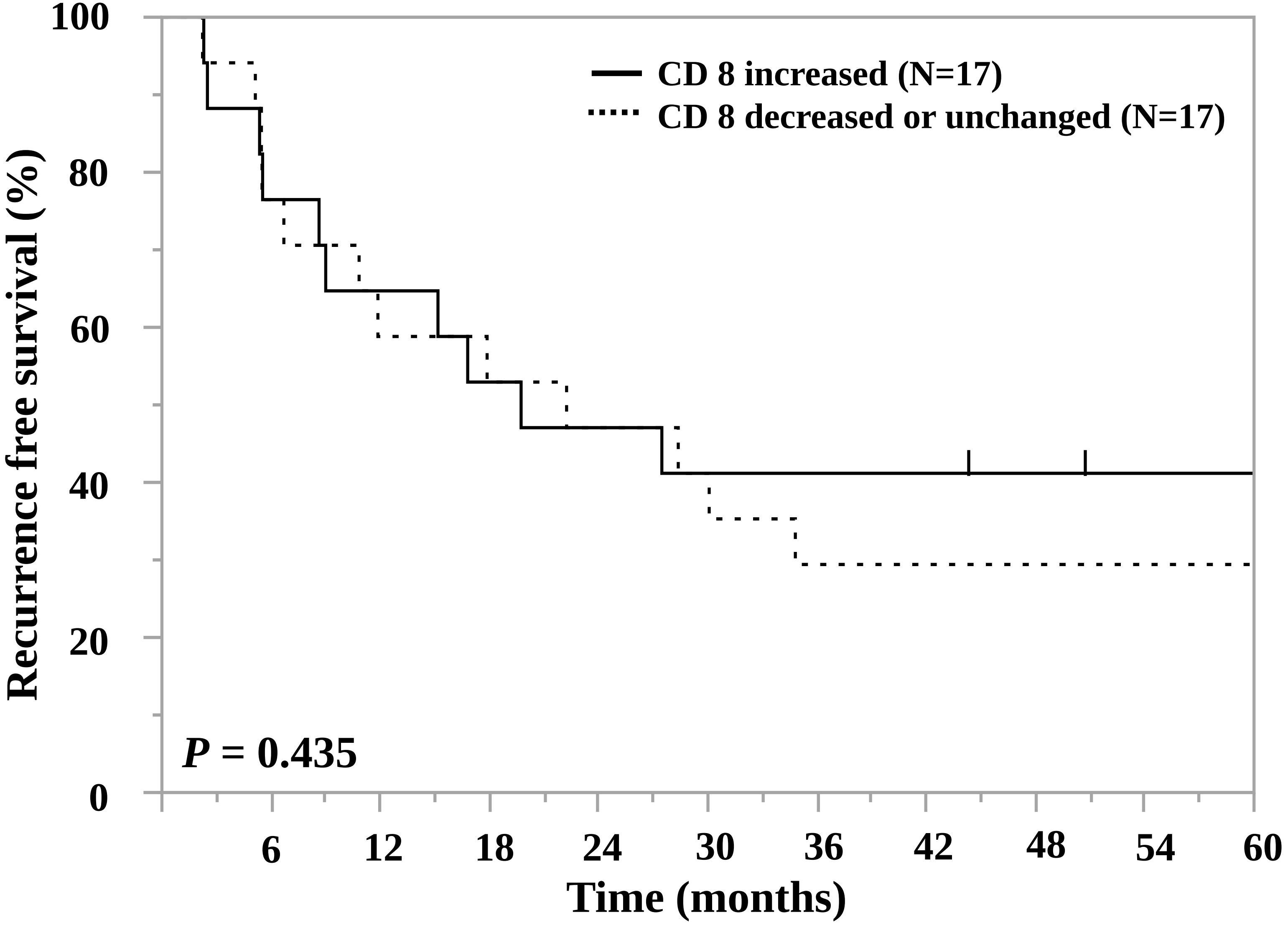

Supplementary Figure 3b

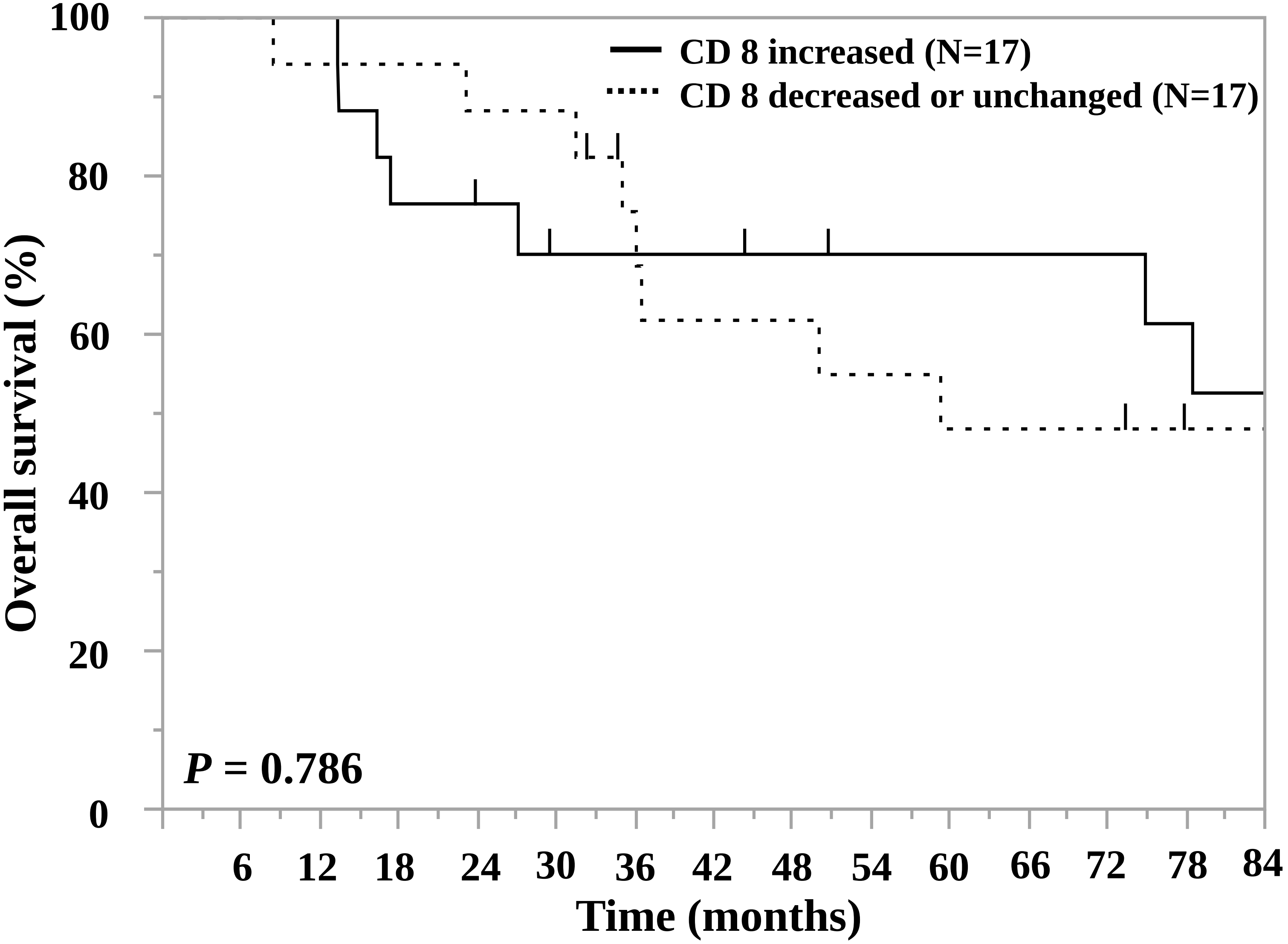

Supplement: Supplementary file 1 — Supplementary Figure [file 41598_2017_11949_MOESM1_ESM.pdf]
